# Interactions between seagrasses and seaweeds during surge nitrogen acquisition determine interspecific competition

**DOI:** 10.1038/s41598-017-13962-4

**Published:** 2017-10-20

**Authors:** Ana Alexandre, Alexandra Baeta, Aschwin H. Engelen, Rui Santos

**Affiliations:** 1 Marine Plant Ecology Research Group, Centre of Marine Sciences (CCMAR), University of Algarve, Gambelas, 8005-139 Faro, Portugal; 2 MARE - Marine and Environmental Sciences Centre, c/o DCV, Faculty of Sciences and Technology, University of Coimbra, Coimbra, Portugal

## Abstract

Seagrasses dominate shallow coastal environments where nitrogen (N) availability in the water column is often sporadic and mainly in the form of pulses. We investigated the N uptake competition between seagrasses and seaweeds through a series of ^15^N surge uptake experiments combining single-species and mixed incubations across ammonium concentrations. N surge uptake rates of seagrasses were 2 to 14-fold higher than those of seaweeds in the majority of combinations, showing that seagrasses are generally in a competitive advantage over seaweeds in N-poor environments with N-pulses. No threshold concentration of ammonium was found beyond which seaweeds performed better than seagrasses. Mixed incubations revealed interspecific interactions that affected rates positively and negatively. Uptake rates obtained in single-species incubations, therefore, cannot always be used to predict the outcome of uptake competition. Only two (*Zostera marina vs*. *Ulva rotundata* and *Zostera marina vs*. *Codium decorticatum*) of the nine combinations tested (*Z*. *marina*, *Z*. *noltei* and *Cymodocea nodosa vs*. *U*. *rotundata*, *C*. *decorticatum* and *Dictyota dichotoma*) were found to enhance macroalgal uptake. Our results showed that the surge uptake capacity of seagrasses represents an important mechanism in their N acquisition strategy that justifies their dominance in shallow oligotrophic environments.

## Introduction

Seagrasses are important habitat-formers and facilitator species that form the basis of complex ecosystems in shallow coastal waters throughout the world^1,2^. Seagrass beds provide food and shelter for a wide variety of organisms, trap suspended organic matter and stabilise soft sediments protecting coastlines from erosions^3^. One of the most relevant ecological functions of seagrasses is nutrient recycling, i.e. the seagrass-mediated processes that cycle and retain nutrients in seagrass beds, such as nutrient acquisition and storage, internal remobilization from older plant parts and rapid mineralization of seagrass-derived organic matter within seagrass beds^4^.

The input of high nitrogen (N) levels in seagrass-dominated systems stimulates the development of macroalgae species. Excessive macroalgal growth causes seagrass displacement^5^, affecting ecosystems dramatically by altering fundamental biogeochemical cycles and species composition^1,6–8^. Macroalgal overgrowth on top of seagrass beds reduces the amount of light available to plants during daytime and O_2_ supply during darkness, leading to loss of fitness and elevated mortality^9–12^. However, in shallow N-poor environments seagrasses dominate as primary producers and biomasses of co-existing macroalgae are usually kept relatively low, suggesting that seagrasses may be better nitrogen competitors than seaweeds up to certain N concentrations. Competition for nitrogen in the sediment has been suggested as the underlying mechanism in observed interactions between the dominant tropical seagrass *Thalassia testudinum* and the native seaweed *Halimeda incrassata* ^13^.

In N-poor environments, such as those that characterise seagrass habitats, the availability of nitrogen in the water column is sporadic and occurs in the form of N pulses. In tidal systems, these N pulses, particular ammonium, may originate from the sediment to the water column as the flood tide first covers the sediments that were exposed to the air during low tide^14,15^. In addition, localised N pulses from microbial remineralisation^16^ or animal excretions^17^ also occur. In this context, surge uptake i.e. enhanced nutrient uptake during short periods (min to h) that often exceeds the required level for growth by several-fold^18^, is an important physiological mechanism that allows species to take advantage of transient peaks of nitrogen. The existence and characterisation of the surge uptake phase has been well described in macroalgae e.g.^19–23^, and in a few seagrass species, like *Z*. *marina* (for ammonium and nitrate), *Z*. *noltei* (for phosphate) and *Amphibolis antarctica* (for ammonium)^24–26^. In *Z*. *noltei*, the ammonium uptake rates by the leaves were 3 to 4 fold higher within the first 30 min of incubation, and within 120 min in the case of nitrate^27^. Surge uptake often occurs in areas where nutrients are limited and may be crucial to sustain growth under nutrient poor conditions^18,19,28^. We hypothesise that in N-poor environments with N-pulses seagrasses have a competitive advantage due to a greater surge uptake capacity relative to seaweeds, allowing them quicker to capture nitrogen and therefore become better N competitors.

We assessed the nitrogen competition dynamics between seagrasses and seaweeds using a variety of seagrasses and co-occurring seaweed species through a series of ^15^N surge uptake experiments combining single-species and mixed incubations, i.e. species were incubated individually and under direct competition. Specifically, we aimed to provide answers to the following questions: i) are seagrasses better than seaweeds in surge uptake, and up to what threshold N concentration do seagrasses perform better, ii) are there any significant interspecific interactions between seagrasses and seaweeds that affect their nitrogen uptake rates and iii) which seagrass vs. seaweed combinations are most prone to macroalgal development, i.e., in which combinations do seaweeds perform better at taking up N? Nitrogen is a fundamental nutrient for seagrass and seaweed growth and one of the most limiting in the marine environment. Ammonium was chosen as the inorganic nitrogen source in these experiments because, in Ria Formosa lagoon, where the study was carried out, N pulses from the sediment to the water column with the incoming tide are mostly in the form of ammonium^15^, and because a compilation of more than thirty published studies comprising eight seagrasses and thirty-four seaweed species showed that ammonium is consistently preferred over nitrate^29,30^.

## Results

**Figure 1:**
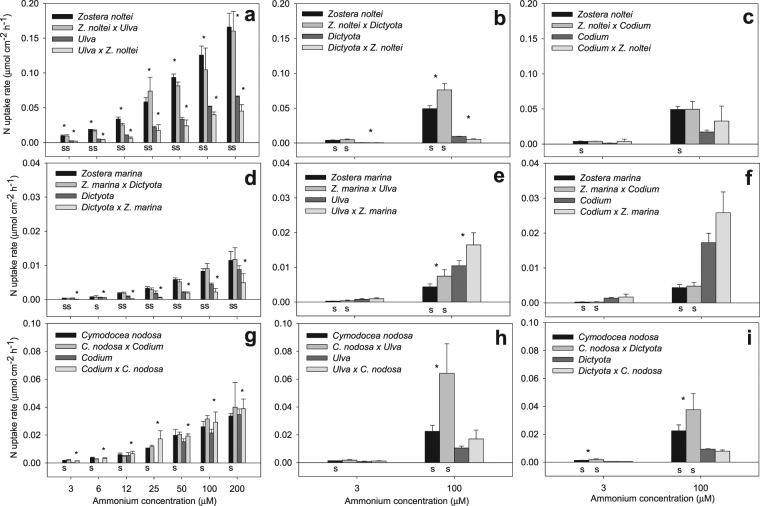
Nitrogen surge uptake rates (µmol cm^−2^ h^−1^) of seagrasses vs. seaweeds: (**a**) *Zostera noltei* *vs.* *Ulva*, (**b**) *Z. noltei vs. Dictyota*, (**c**) *Z. noltei vs. Codium*, (**d**) *Zostera marina vs. Dictyota*, (**e**) *Z. marina vs. Ulva*, (**f**) *Z. marina vs. Codium*, (**g**) *Cymodocea nodosa vs. Codium*, (**h**) *C. nodosa vs. Ulva* and (**i**) *C. nodosa vs. Dictyota*, incubated alone or in competition as a function of ^15^NH_4_Cl concentration (µM). Symbols indicate significant effects of species (S) and treatment (*) (p < 0.05). Values are mean ± standard deviation.

The surge ammonium uptake rates of both seagrasses and seaweeds increased with nutrient concentration both when incubated in isolation or combination with other species (Fig. 1). In general, the effects of species (S), treatment (T) and nitrogen concentration (C) on the ammonium surge uptake of macrophytes were highly significant (Tables 1 and 2). The relative uptake rates of species when incubated in isolation or combination were maintained along the gradient of ammonium concentration in the majority of cases. The uptake rates of the seagrass *Z*. *noltei* were 3 to 14-fold higher than the seaweeds *Ulva* and *Dictyota* irrespective of treatment and N concentration (Fig. 1a,b), and higher than *Codium* only in monospecific incubations at 100 µM N (Fig. 1c). The surge uptake rates of the seagrass *Z*. *marina* exceeded those of seaweeds only in the combination *Z*. *marina vs*. *Dictyota*, where seagrass uptake rates were 4 to 17-fold higher (Fig. 1d). *Z*. *marina* uptake rates were 3 and 6-fold lower than those of *Ulva* and *Codium*, respectively (Fig. 1e,f). N surge uptake rates of *C*. *nodosa* were 2 to 14-fold higher than all seaweed species, except in competition with *Codium* (Fig. 1g) and *Ulva* at 3 µM N (Fig. 1h), where rates were similar.

**Table 1:** Summary of PERMANOVA results for the nitrogen surge uptake rates of each species (S = Species) for the combinations *Zostera noltei vs*. *Ulva*, *Zostera marina vs*. *Dictyota* and *Cymodocea nodosa vs*. *Codium*, measured when species were incubated alone or in competition (T = Treatment) at different ammonium concentrations (C = Concentration). Significant P-values are in bold (p < 0.05).

		*Z*. *noltei vs*. *Ulva*	*Z*. *marina vs*. *Dictyota*	*C*. *nodosa vs*. *Codium*
*Source*	df	P (perm)	P (perm)	P (perm)
S	1	**<0**.**001**	**<0**.**001**	0.130
T	1	**0**.**013**	**0**.**008**	**0**.**041**
C	6	**<0**.**001**	**<0**.**001**	**<0**.**001**
S × T	1	0.541	**0**.**008**	**<0**.**001**
S × C	6	**<0**.**001**	**<0**.**001**	0.917
T × C	6	0.135	**0**.**035**	0.267
S × T × C	6	0.615	0.632	0.111

**Table 2:** Summary of PERMANOVA results for the nitrogen surge uptake rates of each species (S = Species) for the combinations *Zostera noltei vs*. *Dictyota*, *Z*. *noltei vs*. *Codium*, *Zostera marina vs*. *Ulva*, *Z*. *marina vs*. *Codium*, *Cymodocea nodosa vs*. *Ulva and C*. *nodosa vs*. *Dictyota*, measured when species were incubated alone or in competition (T = Treatment) at different ammonium concentrations (C = Concentration). Significant P-values are in bold (p < 0.05).

	*vs.*	*Z. noltei*		Z. marina		*C. nodosa*	
		*Dictyota*	*Codium*	*Ulva*	*Codium*	*Ulva*	*Dictyota*
Source	df	P (perm)	P (perm)	P (perm)	P (perm)	P (perm)	P (perm)
S	1	**0**.**0001**	**0**.**0001**	**0**.**0001**	**0**.**0004**	**0**.**0003**	**0**.**0001**
T	1	**0**.**0001**	0.1506	**0**.**0023**	0.2145	0.0115	0.1625
C	1	**0**.**0001**	**0**.**0001**	**0**.**0001**	**0**.**0001**	**0**.**0001**	**0**.**0001**
S × T	1	**0**.**0001**	0.6825	0.0916	0.5324	0.5296	**0**.**0058**
S × C	1	**0**.**0001**	**0**.**0004**	**0**.**0001**	**0**.**0005**	**0**.**0005**	**0**.**0001**
T × C	1	**0**.**0004**	0.2343	**0**.**0046**	0.2348	**0**.**0166**	0.2412
S × T × C	1	**0**.**0001**	0.8916	0.1093	0.5911	0.6641	**0**.**0072**

Mixed incubations of seagrasses and seaweeds revealed the existence of both negative and positive interactions between macrophytes that affected their individual ammonium uptake rates (Table 3). Negative effects on uptake rates were observed on both competitors (*Z*. *noltei* and *Ulva*), or only on one competitor (on *Dictyota* in the presence of *Z*. *noltei* or *Z*. *marina*). Positive effects on uptake rates of the seagrasses *Z*. *marina* and *C*. *nodosa* were found in the presence of *Ulva*, on *Z*. *noltei* and *C*. *nodosa* in the presence of *Dictyota* and of the seaweeds *Ulva* in the presence of *Z*. *marina* (only at 100 µM) and *Codium* in the presence of *C*. *nodosa* (Table 3). The absence of any interaction between macrophytes was found in the combinations *Z*. *noltei vs*. *Codium* and *Z*. *marina vs*. *Codium* at all ammonium concentrations. Thus, in most cases seagrasses were winners over seaweeds when competing for ammonium surges.

**Table 3:** Summary of the effects of interspecific interactions on the ammonium surge uptake rates of each species. 0 = no effect; − % = percentage decrease relative to the species uptake in monospecific incubation; + % = percentage increase relative to the species uptake in monospecific incubation. Split cells indicate that more than one effect was observed for a specific combination, depending on the nutrient concentration. Values in brackets indicate the specific ammonium concentration at which the effect occurred.

	*Zostera noltei*	*Zostera marina*	*Cymodocea nodosa*	*Ulva*	*Dictyota*	*Codium*
*vs*. *Ulva*	−12%	0 (3 µM)	0 (3 µM)			
		+30% (100 µM)	+65% (100 µM)			
*vs*. *Dictyota*	0 (3 µM)	0	+35%			
	+35% (100 µM)					
*vs*. *Codium*	0	0	0			
*vs*. *Z*. *noltei*				−30%	−40%	0
*vs*. *Z*. *marina*				0 (3 µM)	−50%	0
				+35% (100 µM)		
*vs*. *C*. *nodosa*				0	0	+50%

## Discussion

Surge uptake is an important component of the uptake process as it may determine the competitive ability of a species to obtain the necessary nutrients in environments where nutrient concentrations generally are low. We showed here that seagrasses exhibit a remarkable uptake capacity of ammonium surges, which exceeded that of co-occurring seaweeds by several-fold in the majority of combinations. All seagrass species studied were able to take up ammonium more rapidly than seaweeds, except *Z*. *marina* when combined with *Ulva* and *Codium*. No threshold concentration of ammonium was found beyond which seaweeds performed better than seagrasses, suggesting that competition between seagrasses and seaweeds for ammonium surges is determined by the species-specific surge uptake rate rather than by the surge concentration.

Mixed species incubations revealed the existence of interactions between seagrasses and seaweeds. Both positive and negative effects on uptake rates were observed relatively to the rates of monospecific incubations, determining the competitive uptake winner at specific combinations. This important finding shows that the uptake rates of macrophytes, when incubated individually, cannot always be used to predict the outcome of uptake competition between seaweeds and seagrasses. Similar findings were also reported in a competition study of macroalgae *vs*. phytoplankton^31^, where the nutrient uptake dynamics under competitive conditions could not be predicted using individual nutrient uptake parameters. To our knowledge, our work is the first that directly measures competitive dynamics of nutrient uptake rates between seagrasses and macroalgae.

The ability of seagrasses to quickly remove nitrogen as it becomes available reflects their adaptation to environments where nutrient concentrations are typically very low but where pulses of nutrients normally occur^32,33^. In N-limited environments, where competition for the nutrient is high, seagrasses may be in a competitive advantage over seaweeds since they can explore short-lived pulses of nitrogen from the water column more efficiently, thus increasing their ability to maintain growth in environments with fluctuating N concentrations. Our results suggest that the surge uptake capacity of seagrasses represents an important mechanism in their N acquisition strategy that favors their survival and dominance in shallow oligotrophic environments.

In this study, the leaves and roots of seagrasses were incubated in the same compartment at the same concentrations, and the uptake by both plant parts was integrated as a whole-plant uptake, which we compared with the seaweed uptake. Even though ammonium-rich sediments are often considered the primary source of nitrogen for seagrasses, previous studies showed that the uptake of ammonium through the roots does not contribute significantly to the overall seagrass N acquisition because root uptake is typically much lower compared to those by the leaves in several seagrass species^34^ and references therein. Thus, the whole-plant uptake rates of ammonium obtained in this study by incubating leaves and roots in the same concentration should not vary much from uptake rates obtained from incubating both plant parts separately at different concentrations. To completely unveil the hypothesis currently formulated that seagrasses are more efficient than seaweeds in nutrient uptake under low nutrient concentrations^4^, and may thus prevent macroalgal development when some nutrient threshold is exceeded, the long-term uptake rates of seagrasses *versus* seaweeds must also be analysed. However, care should be taken to address this hypothesis as nutrient uptake studies have been mostly done in single-species incubations. As we showed here, in some specific cases the uptake rates can be significantly altered in the presence of other species.

The observed interspecific interactions between seagrasses and seaweeds, which affected positively or negatively their individual ammonium uptake rates, may be explained by a specific limitation of other essential elements (e.g. phosphorus and carbon) that interfere with the uptake of ammonium. For example, *Z*. *noltei* has been shown to use the dissolved organic carbon excreted by *Ulva* to enhance growth^35^, something that could have benefit some of the seagrass species in our experiments (*Z*. *marina* and *C*. *nodosa*). More complex interspecific interactions may be expected if seagrasses are incubated with multiple species of seaweeds, and vice-versa, as in the natural environment. However, the hypothesis that a limitation by essential nutrients may affect the ammonium uptake rates between seagrasses and seaweeds must be experimentally tested. Another possible explanation is allelochemical-mediated interference. Allelopathy, i.e. the release of chemical substances by one plant eliciting positive or deleterious responses on another^36^, is known to be involved in interspecific competition between several aquatic macrophytes^37–40^. The chemicals released can affect numerous physiological processes in the target species, such as growth, photosynthetic performance, enzymatic activity and nutrient uptake^39,41^. In the present study, the ammonium uptake rates of the seaweeds *Ulva* and *Dictyota* were negatively affected by the presence of the seagrasses *Z*. *noltei* and *Z*. *marina* (except in the combination *Ulva* vs. *Z*. *marina*). We are not aware of any studies reporting allelochemical effects of seagrasses on macroalgae, but water soluble extracts of leaves of *Zostera* species contain several inhibitory substances, such as zosteric acid, flavonoids and phenolics^42^, which negatively affected the development and photosynthetic carbon uptake of epiphytic diatoms^43,44^, as well as growth of microalgae and marine bacteria^45^. A negative effect of macroalgae on the N uptake rates of seagrasses was found only in the combination *Z*. *noltei vs*. *Ulva*. *Ulva* species are well known for their strong allelopathic inhibitory effects on micro- and macroalgae^40,46–48^ but effects on seagrasses have not been reported. Although by definition beneficial allelopathic effects may also occur, only a few studies reported such effects, and they were mainly observed in crop plants e.g.^49^. Nonetheless, in our study, we found positive seagrass-seaweed interactions in a large number of combinations, where the N uptake rates of at least one species increased relatively to those of monospecific incubations. As a result, the collective nitrogen uptake in mixed incubations was higher than the total N uptake in monospecific incubations. This is an interesting result and suggests that macrophyte diversity may increase the total nitrogen uptake capacity of seagrass-dominated ecosystems as reported for seaweeds in tidal rock pools^50^.

The winners for ammonium uptake in different seagrass-seaweed combinations depend not only on the surge uptake capacity of each species but also on their interactions. Consequently, one seagrass species may be a winner in one specific combination but not in others. For example, the seagrass *Z*. *marina* was the uptake winner when combined with *Dictyota*, but not when combined with *Ulva* or *Codium*, irrespective of the ammonium concentration. The combinations *Z*. *marina vs*. *Ulva* and *Z*. *marina vs*. *Codium* were the most prone to macroalgal development because the surge uptake rates of the seagrass were always lower than the seaweeds. *Z*. *marina* is one of the most threatened seagrass species worldwide^51^, mainly due to the N enrichment of coastal habitats^52^, and is the most endangered of the three existing species in Ria Formosa lagoon^53^. It is possible that the global decline of *Z*. *marina* may be related to its lower surge uptake capacity relative to seaweeds. In the specific case of Ria Formosa, *Z*. *marina* appears to be close to a light-mediated ecophysiological threshold being less competitive for light than the sympatric seagrass *Cymodocea nodosa* ^54^.

In conclusion, this study clearly shows that seagrasses can compete with seaweeds during surge uptake and thus prevent opportunistic macroalgae blooms in N-poor environments with high, transient ammonium inputs irrespective of their concentration. Significant species-specific interactions may affect the seagrass-seaweed competitive outcome. Although no interspecific interactions were observed in most of the combinations, positive effects were mostly observed over the uptake rates of seagrasses in the presence of seaweeds.

## Methods

### Site description and plant collection

Ria Formosa is a mesotidal coastal lagoon located in South Portugal (37°01′N, 7°51′W). In this system, *Zostera noltei* is the most abundant seagrass species, developing extensive meadows along the intertidal mudflats and major contributor to the lagoon’s metabolism^55^. The subtidal areas of the lagoon are occupied by the seagrass species *Zostera marina* and *Cymodocea nodosa*. Bloom-forming macroalgae co-occur with seagrasses in the lagoon. *Ulva* species, mostly *U*. *rotundata* cover mostly the intertidal areas occupied by the seagrass *Z*. *noltei*, but also settle over *Z*. *marina* and *C*. *nodosa* meadows. Other seaweeds also thrive, namely species of *Dictyota dichotoma* and *Codium decorticatum*, although these species develop more frequently in the subtidal areas of the lagoon. Ammonium and nitrate concentrations in the water column are usually less than 5 µM due to a high water exchange with the adjacent ocean during each tidal cycle^56^, but ammonium pulses (∼10 µM) from the sediment to the water column occur with the incoming tide^15^. Ammonium concentration in the sediment pore water is higher (12–38 µM), whereas nitrate concentration is almost negligible (0.2–0.9 µM)^57^. Macroalgae (*U*. *rotundata*, *D*. *dichotoma* and *C*. *decorticatum*) and seagrass species (*Z*. *noltei*, *Z*. *marina* and *C*. *nodosa*) were collected during the autumn and winter of 2012. In the laboratory, seagrass roots were carefully cleaned of adherent sediment and leaves were cleaned of epiphytes. The species were kept separately in aquaria with seawater from the collection site for two days to acclimate to the experimental conditions (seawater temperature of 14 °C and light intensity of 300 µmol quanta m^−2^ s^−1^).

### Experimental procedure

In a first experiment, the competitive dynamics of ammonium uptake between seagrasses and seaweeds was studied using three different combinations of co-occurring species in the lagoon: *Z*. *noltei vs*. *Ulva*, *Z*. *marina vs*. *Dictyota* and *C*. *nodosa vs*. *Codium*. In each combination, the ammonium uptake rate of each species was assessed by incubating them separately and in competition in nitrogen-free artificial seawater (salinity of 35‰, pH of 8.24) enriched with ^15^NH_4_Cl (atom % = 98, Sigma) at seven different concentrations (3, 6, 12, 25, 50, 100, 200 µM) for 30 min. Incubations were performed in triplicate for each nutrient concentration.

In a second experiment, N competition dynamics was studied in another six different combinations of seaweeds and seagrasses (*Z*. *noltei vs*. *Dictyota*, *Z*. *noltei vs*. *Codium*, *Z*. *marina vs*. *Ulva*, *Z*. *marina vs*. *Codium*, *C*. *nodosa vs*. *Ulva and C*. *nodosa vs*. *Dictyota*), so that all possible combinations of the three seagrass and seaweed species were tested. The ammonium uptake rate of each species was assessed at low (3 µM) and high (100 µM) ammonium concentrations by incubating species separately or in competition as described above.

The incubation conditions were identical in the two experiments. The media were constantly stirred to decrease the thickness of the boundary layer and to ensure a homogeneous distribution of the isotopic labels. The biomass to volume relationship of the incubations was previously determined in preliminary experiments to ensure that the ammonium concentrations remained constant throughout the specific incubation period (i.e. no substantial change in the nutrient concentration occurred), preventing any significant ammonium limitation that could interfere in the rate of nutrient uptake. When in competition, one single seagrass module (i.e. shoot with respective rhizome and roots) and its equivalent fresh weight of seaweed were collectively incubated to eliminate the possibility that any existing interspecific interactions that could affect the uptake rates was due to differences in biomass between the two species. In single-species incubations, two seagrass modules and the equivalent fresh weight of seaweed were incubated separately. Species were immersed in 1 L of nitrogen-free artificial seawater (salinity of 35‰, pH of 8.24). Seawater was prepared using MilliQ ultrapure water with a pH of 5. The pH of the seawater solution was adjusted to 8.24 using HCO_3_^−^, which also provided the media with a source of inorganic carbon. The fresh weight of one seagrass module of *Z*. *noltei* was on average 0.12 ± 0.07 g (0.02 ± 0.01 g dry weight). One module of *Z*. *marina* averaged 1.06 ± 0.90 g fresh weight (0.19 ± 0.16 g dry weight) while one module of *C*. *nodosa* averaged 1.13 ± 0.57 g FW (0.20 ± 0.1 g dry weight). The aboveground: belowground biomass ratio was 1.47 for *Z*. *noltei*, 4.83 for *Z*. *marina* and 0.76 for *C*. *nodosa*. All experiments were performed in a walk-in culture chamber at constant temperature (14 °C) and light intensity (300 µmol quanta m^−2^ s^−1^). This light intensity has been shown to saturate, or nearly saturate, photosynthesis in virtually all studied species^58–61^. At the end of the incubation, tissues were removed from the media, seagrass leaves were immediately separated from the rhizomes and roots, and all tissues were briefly rinsed with deionised water to remove adherent salts and label (^15^N). Tissues were dried at 60 °C for 48 h and reduced to a fine powder. Total nitrogen content and the percentage of ^15^N of dried tissues were determined using a Flash EA 1112 Series elemental analyser coupled on line via a Finningan conflo II interface to a Thermo delta V S mass spectrometer. Precision in the overall preparation and analysis was better than 0.2‰. ^15^N background levels of seagrass leaf, root and rhizome tissues and seaweed tissue were measured as controls (n = 5).

Though in nature the rhizosphere of seagrasses can be anoxic, in these experiments we incubated whole plants in an oxygenated medium. Previous experiments showed no effects of rhizosphere oxygenation on the ammonium uptake rates of leaves and roots of *Z*. *noltei* ^34,62^, which was confirmed for *Z*. *marina* in preliminary experiments of the present study.

### Data analysis

^15^N enrichment (%) of tissues after incubations were calculated by subtracting the post-incubation ^15^N levels from the initial background levels, which was multiplied by the total nitrogen content of the tissue (g) and then divided by its weight (g dry weight). For seagrasses, the uptake rates were expressed as whole-plant uptake rates and were calculated by the sum of the uptake rates of leaves and roots calculated using the surface area of the respective plant part and then divided by the sum of the surface areas of the plant parts. Because nutrient uptake during the surge phase is diffusive, the extent of the surge uptake rate is expected to be a direct function of the number and activity of sites available for the nutrient transport into the cells at the plant surface^63^. We, therefore, believe that, when evaluating interspecific competition during the surge phase, uptake rates should be expressed per surface area units rather than per biomass units. The latter would be more appropriate when comparing uptake rates during the subsequent internally controlled phase, i.e. when uptake rates are controlled by the rate of nutrient assimilation in the cells^22^. All incubated tissues were photographed, and their surface areas were calculated using the software Image J^64^. Significant differences in the nitrogen uptake rates of seagrass and seaweed species (S) incubated alone or in competition (T) at different nitrogen (N) concentrations were analysed for all combinations of species tested using a permutational analysis of variance^65^, using the PERMANOVA module^66,67^ within Primer 5 software^68^, with three fixed factors: 1) species, with two different levels: seagrass and seaweed, 2) treatment, also with two levels: alone and in competition; and 3) N concentration, with seven different levels: 3, 6, 12, 25, 50, 100 and 200 µM in the first experiment, and two levels: 3 and 100 µM in the second experiment. This method does not require implicit assumptions about the underlying distribution (i.e. normality) or spread (i.e. variance) of the data, hence does not assume either normality or homoscedasticity. Permutation of residuals under a reduced model, with 9999 permutations on a data matrix of average distance measures was performed as recommended to test distance based homogeneity of dispersion, main effects and pair-wise tests on significant factors/interactions. In case the number of unique permutations was lower than 100, Monte Carlo permutations(9999) p-values were used.

### Data availability statement

The datasets generated during and/or analysed during the current study are available from the corresponding author on reasonable request.
